# Tribological performance of Graphene/Carbon nanotube hybrid reinforced Al_2_O_3_ composites

**DOI:** 10.1038/srep11579

**Published:** 2015-06-23

**Authors:** Bahareh Yazdani, Fang Xu, Iftikhar Ahmad, Xianghui Hou, Yongde Xia, Yanqiu Zhu

**Affiliations:** 1 College of Engineering, Mathematics and Physical Sciences, University of Exeter, Exeter EX4 4QF, UK; 2 Department of Mechanical, Materials and Manufacturing Engineering, The University of Nottingham, Nottingham, NG7 2RD, UK; 3 Center of Excellence for Research in Engineering Materials, Advanced Manufacturing Institute, King Saud University-Riyadh 11421 - P.O. Box 800 - Kingdom of Saudi Arabia

## Abstract

Tribological performance of the hot-pressed pure Al_2_O_3_ and its composites containing various hybrid contents of graphene nanoplatelets (GNPs) and carbon nanotubes (CNTs) were investigated under different loading conditions using the ball-on-disc method. Benchmarked against the pure Al_2_O_3_, the composite reinforced with a 0.5 wt% GNP exhibited a 23% reduction in the friction coefficient along with a promising 70% wear rate reduction, and a hybrid reinforcement consisting of 0.3 wt.% GNPs + 1 wt.% CNTs resulted in even better performance, with a 86% reduction in the wear rate. The extent of damage to the reinforcement phases caused during wear was studied using Raman spectroscopy. The wear mechanisms for the composites were analysed based on the mechanical properties, brittleness index and microstructural characterizations. The excellent coordination between GNPs and CNTs contributed to the excellent wear resistance property in the hybrid GNT-reinforced composites. GNPs played the important role in the formation of a tribofilm on the worn surface by exfoliation; whereas CNTs contributed to the improvement in fracture toughness and prevented the grains from being pulled out during the tribological test.

Structural ceramics are becoming a new class of materials for advanced engineering applications due to the limitation of polymers and metals in extreme environments (e.g. high temperature/pressure, nuclear radiation and chemicals). Particularly, the friction and wear behaviour of ceramic-based composites is of great importance for contact-mechanical (e.g. bearing, valves, nozzles, armour, and prostheses) and protective coating applications. Accordingly, a wide range of reinforcements such as fibres^1^,^2^, particles^3^, and carbon nanotubes (CNTs)^4^,^5^,^6^,^7^, have been incorporated into different ceramic matrices to fabricate composites, with the main objective to curtail the intrinsic brittleness and widen their applications.

The recently discovered graphene has shown exceptionally high mechanical (Young’s modulus of 1.0 TPa), electrical and thermal properties, which make it one of the most promising reinforcements for ceramic matrix composites (CMCs)^8^,^9^,^10^,^11^. This 2-dimetional (2D) sheet of carbon has high surface areas compared to graphite, carbon black and CNTs, thus a small loading (less than 1.0 vol%) in a matrix may lead to large improvements in the composite properties. In the case of CNTs, another wonder materials for decades, a higher concentration (1 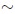 10 vol%) was generally required for toughening and strengthening of ceramics^12^,^13^.

Moreover, graphene is a good candidate for solid lubrication that reduces the friction force between contact surfaces at micro- and nano-scale. A nanometer-thick surface layer of hard, strong, and lubricating graphene on ceramic grains may lead to a huge improvement in the contact-damage resistance. In fact, some tribological properties of graphene nanoplatelets (GNPs) reinforced ceramic nanocomposites with obvious reductions in wear volume have also been documented^14^,^15^,^16^,^17^. Polymer-based composites reinforced with graphene oxide and CNTs also showed significant reductions in the wear rate^18^,^19^. Shen *et al* reported that epoxy reinforced with hybrid graphene oxide (GO) and CNT obtained 40% additional reduction in specific wear rate compared with those containing only CNTs, due to the improved CNT dispersion in the presence of GO^19^.

In our previous work, we also applied a hybrid of CNTs and GNPs (GNTs) into an Al_2_O_3_ matrix and achieved significant improvements in terms of different mechanical properties^11^. In this paper, the wear resistant performance of the Al_2_O_3_-GNT nanocomposites with various GNT contents, under various sliding loads, will be our main research focus. We will report the excellent low wear rate, analyse and discuss the wear mechanism of this novel hybrid reinforcement.

## Materials and Methods

### Materials and characterization techniques

**Figure 1:**
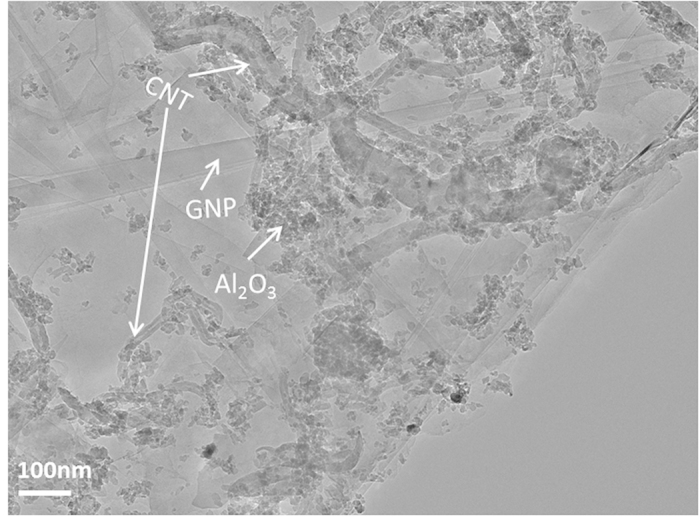
TEM image of the Al_2_O_3_-GNT powder mixture, prior to hot-press sintering, showing the uniform dispersion of the constituents.

GNPs (ABCR GmbH & Co, Germany, 6-8 nm thick × 5 μm wide) and CNTs (Tsinghua University, China, average outer diameter of 40 nm), as shown in Fig. 1, were highly dispersed within Al_2_O_3_ nanopowder (40 nm gamma-Al_2_O_3_ nanoparticles provided by Sasol, Germany) by a unique environmental friendly wet mixing technique assisted by a probe-sonication, and the mixed powders were subsequently hot-pressed at 1650°C under 40 MPa under Ar atmosphere, as described previously^11^.

The densified square samples (50 × 50 × 2.5 mm) were categorized according to the concentration of reinforcing constitutions (GNP and CNT) are listed in Table 1, designated as S_X-Y_, where X represents the GNP wt% and Y for the CNT wt%. The Archimedes method was used to measure the densities of the samples (using distilled water), and the relative densities were calculated by dividing the apparent density by the theoretical density. For consistency, 3.97 g/cm^3^, 1.85 g/cm^3^ and 2.21 g/cm^3^ were used as the theoretical densities for Al_2_O_3_, CNTs and GNPs, respectively^11^. Structural features of the worn surfaces were examined using SEM (Hitachi S3200N), and X-ray diffraction patterns were obtained on a Bruker D8 Advance XRD machine to analyse the crystalline phase features of all samples. Raman spectroscopy (Renishaw RM1000) was also used to evaluate the structural changes of both GNPs and CNTs before and after the wear testing.

### Mechanical properties

Vickers hardness testing was performed using a 5 kg load for 15 s, and an average of five equally spaced indents was recorded for each sample. The flexural strength (*σ*_f_) was measured using the three-point bending technique, and the size of specimens was 20 mm (length) × 2 mm (breadth) × 2.2 mm (height). The bending span and the loading speed for the flexural strength testing were 16 mm and 0.5 mm/min, respectively. At least 4 bars were tested for each sample. The fracture toughness (*K*_IC_) was evaluated using the single edge notched beam (SENB) method. Four notched specimens with identical dimensions of 14 mm (length) × 2.6± 0.20 mm (breadth) × 2.2 ± 0.15 mm (height) were prepared for each test using a diamond blade. The ratio of the notch depth to the specimen height (*a*/*W)* was 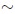
0.45–0.55 according to the ASTM standard (ASTM C1421–99)^20^. Three-point bending test was carried out using a bending span of 8 mm and a loading speed of 0.01 mm/min, to achieve slow crack propagation.

The fracture toughness values were calculated using the maximum force, evaluated from the force–deflection curve, and the specimen dimension using the following equation^7^,^11^.


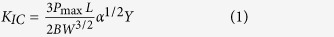


where Y is the minimum of geometrical compliance function, and *B* and *W* are the width and height of specimens, respectively.

The brittleness index *(BI)* was measured using equation (2), where *H* represents the hardness and *K*_*IC*_ for the fracture toughness of the material^21^.


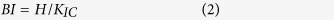


### Wear testing technique

The friction and wear experiments were performed on a wear testing machine using a ball-on-disc configuration in linear reciprocating mode^22^,^23^. A Si_3_N_4_ ball of ϕ 6.3 mm (produced by Dejay Ltd., UK) was used against the pre-polished flat sample surfaces. The tests were carried out at different loads of 5 N, 15 N, 25 N and 35 N, at a fixed sliding speed of 10 mm/s with a reciprocating stroke of 10 mm and duration of 120 min. The friction force transferred to a load cell was recorded throughout the tests. The cross-sectional areas of wear tracks of the samples were measured using a TALYSURF CLI 1000 profilometer (Taylor/Hobson Precision, UK), and the volume losses of the samples were calculated using the TalyMap Universal software. The specific wear rates were calculated using equation (3)^24^.


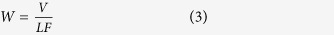


where *L* is the sliding distance, *F* is the applied load, and *V* the wear volume.

## Result

### Mechanical properties and strucrural features of Al_2_O_3_-GNT composites

Wear property in ceramic materials is closely attributed to their mechanical properties, therefore Table 1 summarises the chemical composition, relative densities and their corresponding mechanical properties of different Al_2_O_3_-GNT nanocomposites and pure Al_2_O_3_ prepared by hot-press. Sample identified with S_0.5-0_ exhibited a 58% improvement in the fracture toughness due to various toughening mechanisms, as discussed previously^11^. However, the GNP anchoring around Al_2_O_3_ grains played the most important role in toughening the composites. Increasing the GNP content up to 2 wt% deteriorated the mechanical properties, due to the debonding phenomenon of overlapped large GNPs^11^.

It has been shown that the brittleness index (BI), defined as the H/K_IC_ ratio of a material which reflects the combined responses of the material, is a better parameter for the quantification of wear resistance than taking either the H or the K_IC_ alone separately^21^. Accordingly, a lower hardness combined with higher fracture toughness (lower BI) will make the material more tolerant to damage during wear. Thus BI values of various samples are also presented in Table 1.

**Figure 2:**
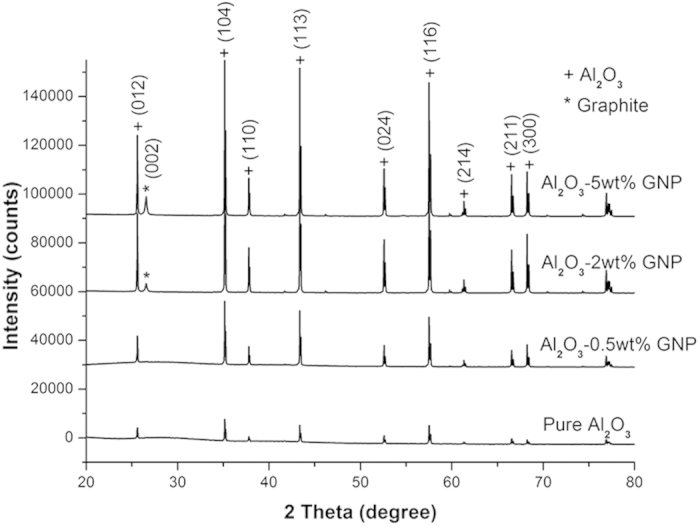
XRD patterns of the Al_2_O_3_ with and without GNP additions.

The typical peaks of α-Al_2_O_3_ were shown in all nanocomposites samples in their XRD profiles (Fig. 2), without any detectable carbide phases, indicating no significant reactions between the GNPs and the Al_2_O_3_ during the consolidation process. At high GNP contents, an additional peak appeared at 26.3° for S_2-0_, which became more detectable for S_5-0_. This peak corresponds to the basal plane in crystalline graphite, and its appearance suggests that the GNPs remained undamaged and kept their integrity after the hot-pressing. When the GNP contents are higher than 2%, the platelet structure makes the reflection from the (002) planes dominant and is easily detectable by XRD.

### Tribological results

**Figure 3:**
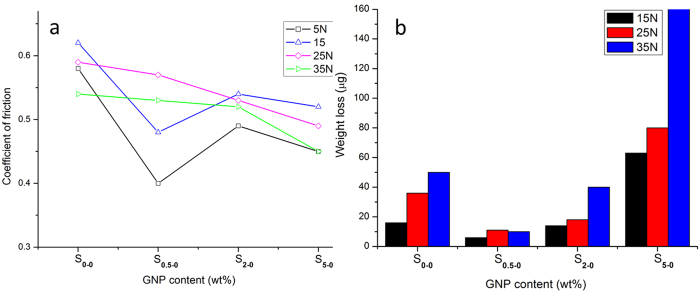
Effect of GNP contents on the (**a**) coefficient of friction and (**b**) wear loss of Al_2_O_3_-GNP composites tested at different loads.

Figure 3a represents the variation of coefficient of friction (COF) as a function of the GNP contents, at different loading conditions. It is clear that the COF trend is different at lower applied loads (5 N and15 N) from that at higher applied loads (25 N and 35 N). Under lower applied loads (5 N and 15 N), there is a minimum point in the COF value for sample S_0.5-0_, with a 23% reduction using 15 N compared with the pure Al_2_O_3_ sample. When the load was increased to 25 N and 35 N, the COF continues to decrease with the GNP content increases. The different responses to varied applied loads during wear is related to the machanical properties of the samples, which will be discussed later after the wear track analysis in section 4.

The weight loss varations as a function of GNP content is shown in Fig. 3b. In all samples, the weight loss increased with increasing the sliding load. The lowest value for weight loss was observed for S_0.5-0_, with a 60%, 70% and 80% reduction compared with S_0-0_, under the sliding loads of 15 N, 25 N and 35 N, respectively. These results shows that, under low GNP contents up to 2 wt%, the composites outperformed the pure Al_2_O_3_; whilst at high GNP contents of 5 wt%, the wear performance of the composite deteriorated.

### Wear properties and microstructures

**Figure 4:**
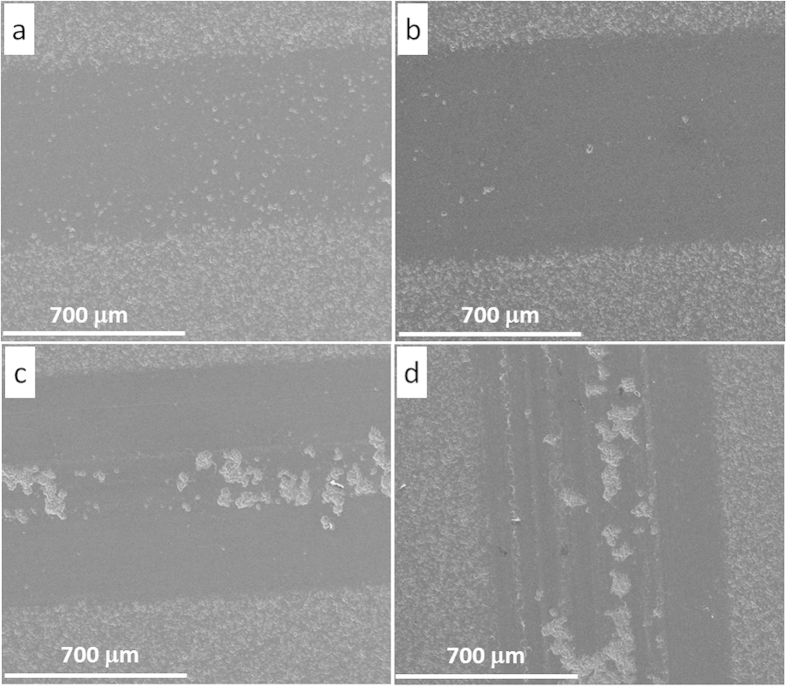
SEM images of the wear track of pure Al_2_O_3_ (S_0-0_) under various sliding loads. (**a**) 5 N, (**b**) 15 N, (**c**) 25 N, and (**d**) 35 N.

Figure 4 shows the SEM images of the wear track of pure Al_2_O_3_ under four different sliding loads and the texture of worn surfaces clearly depicts the load-dependant wear behaviour in pure Al_2_O_3_ samples. As the sliding load increased, the wear track became wider. Moreover, the lower sliding loads (5 N and 15 N) produced relatively smoother wear track surfaces, with very small amount of grains being pulled out. However, the 25 N sliding load caused a larger area of grain pull-out (Fig. 4c), and the 35 N load led to even severe damage to the wear surfaces, with traces of wear groves and large residue debris on the surface, as shown in Fig. 4d. Such Al_2_O_3_ grain pull-outs under 25 N and 35 N sliding loads produced a large amount of wear debris which in turn resulted in abrasive sliding wear.

**Figure 5:**
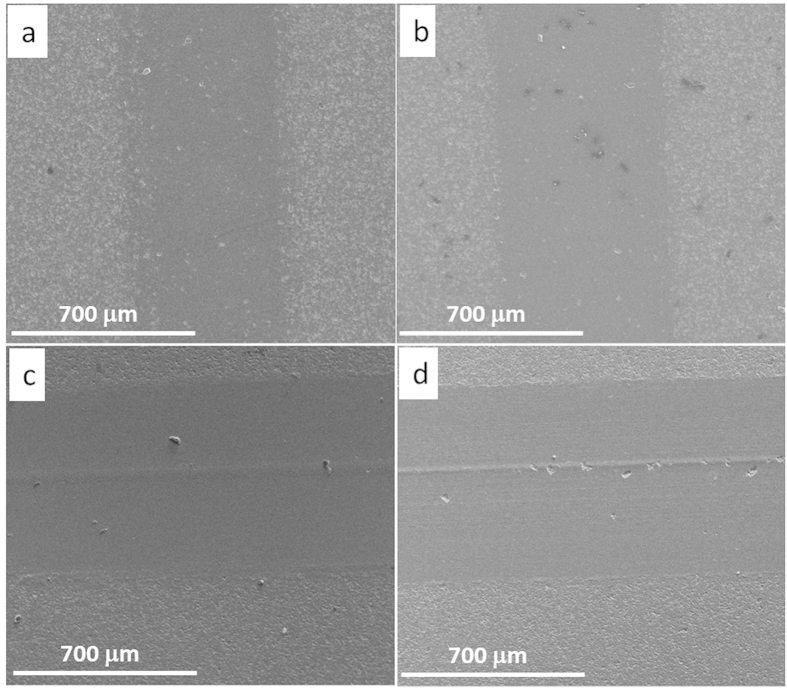
SEM images of the wear track of sample S_0.5-0_, under various sliding loads. (**a**) 5 N, (**b**) 15 N, (**c**) 25 N, and (**d**) 35 N.

The SEM images of wear tracks of S_0.5-0_ under four different sliding loads are shown in Fig. 5, which are entirely dissimilar to wear tracks of pure Al_2_O_3_, as shown in Fig. 4. Under 25 N sliding load (Fig. 5c), there are no grains being pulled out for S_0.5-0_, whilst such a sliding load caused severe damage to the worn surface of the pure Al_2_O_3_ (Fig. 4c). Based on these detailed pictorial worn surfaces, it seems that the higher fracture toughness of S_0.5-0_ compared with pure Al_2_O_3_, as summarised in Table 1, strengthened the grain boundaries and stopped the grains being pulled out at low sliding stresses and strains. Even under 35 N load for S_0.5-0_ (Fig. 5d), the grain pull-out damage was minimal compared with the pure Al_2_O_3_ under the same sliding load (Fig. 4d). However, the grove traces in Fig. 5c,d indicate a deformation controlled wear behaviour under 25 N and 35 N sliding loads. This wear behaviour change probably weakened the lubricating merit of the GNPs in the composites under coarse wear, hence the reduction in COF for S_0.5-0_ under higher sliding loads is not so obvious, as shown in Fig. 3a. In this context, the 15 N sliding load was kept constant for comparing the wear performance of various Al_2_O_3_-GNT composites.

**Figure 6:**
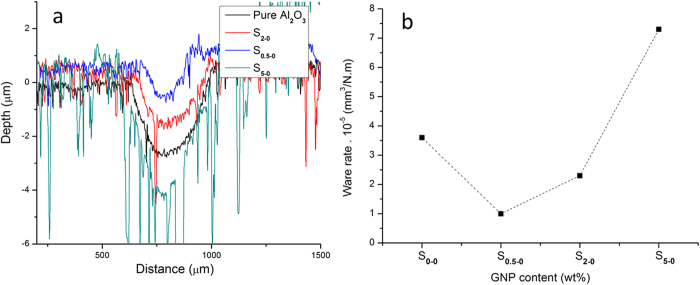
Wear track profiles (**a**) and wear rates (**b**) of the pure Al_2_O_3_ and Al_2_O_3_-GNP composites.

The wear track profiles of pure Al_2_O_3_ and Al_2_O_3_-GNP composites are shown in Fig. 6a, and the wear rates were calculated uisng equation (3) and plotted in Fig. 6b. According to the wear track profile (Fig. 6a), the GNP contents played a critical role in the tribological properties. The worn volume decreased with increased GNP contents, up to 2 wt%, however adding 5 wt% GNPs deteriorated the wear resistant property and drastically increased the worn volume (Fig. 6a), and the wear rate (Fig. 6b). It is clear that adding 0.5 wt% GNPs into the Al_2_O_3_ matrix led to the biggest improvement in the wear resistance, resulting in over 70% reduction in the wear rate, benchmarked against the pure Al_2_O_3_ (Fig. 6b). Such a huge wear resistant improvement in the S_0.5-0_ also matched well with the previously confirmed lowest COF of S_0.5-0_ amongst other GNP single phase reinforced composites (Fig. 3a), and the lowest wight loss (Fig. 3b).

**Figure 7:**
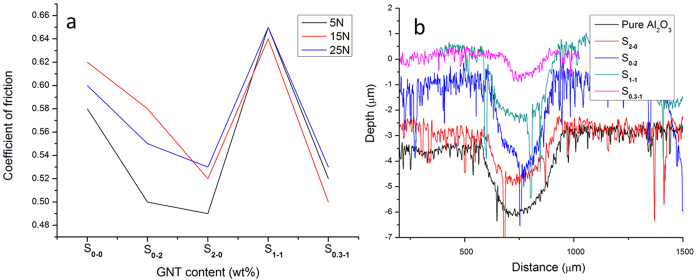
A comparison of the coefficient of friction (**a**), and the wear track profiles (**b**) of the pure Al_2_O_3_ and Al_2_O_3_-GNT composite samples.

After adding the hybrid GNT reinforcement into the Al_2_O_3_ matrix, their COF and wear track profiles of the Al_2_O_3_-GNT composites are exhibited in Fig. 7a,b respectively, against the pure Al_2_O_3_. Sample S_0.3-1_ showed a 20% reduction in the COF (Fig. 7a) and a 74% reduction in the worn volume, compared with the pure Al_2_O_3_, under the 15 N sliding load (Fig. 7b). The large improvements in the wear resistant properties are in line with the excellent mechanical properties for both S_0.5-0_ and S_0.3-1_ (Table 1). Conversely, the mechanically weak nanocomposites, S_5-0_, exhibited poor wear resistance (Fig. 6).

**Figure 8:**
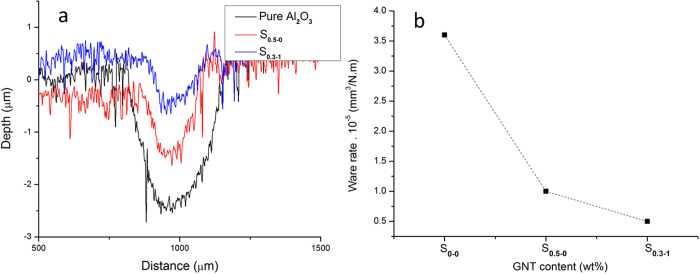
A comparison of the S_0.5-0_ and S_0.3-1_ performance against pure Al_2_O_3_ (**a**) wear track profiles and (**b**) wear rates.

Having compared the wear tracks of S_0.5-0_ and S_0.3-1_, we realise that S_0.3-1_ outperformed S_0.5-0_ marginally, with a slightly smaller worn volume and lower wear rate (Fig. 8a,b) and their wear rates dropped by 70% and 86% for S_0.5-0_ and S_0.3-1_ respectively, against Al_2_O_3_. The hybrid reinforcement shows huge potentials in tailoring the wear properties of the composites.

Further, we compared the above excellent tribological results of the composites with their BI values listed in Table 1. The BI value of S_0.3-1_ is slightly lower than that of S_0.5-0_, which means the former has better machinary properties than S_0.5-0_. This further confirms the advantages of applying hybrid reinforcement in ceramic composites.

## Wear mechanism discussion

**Figure 9:**
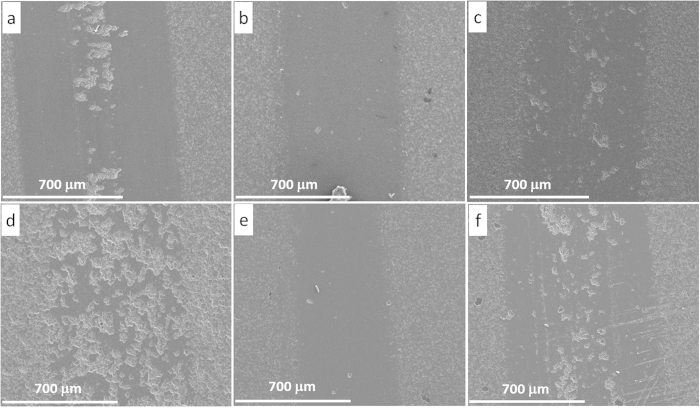
SEM images of wear tracks of (**a**) pure Al_2_O_3_, (**b**) S_0.5-0_, (**c**) S_2-0_, (**d**) S_5-0_, (e) S_0.3-1_ and (f) S_1-1_.

**Figure 10:**
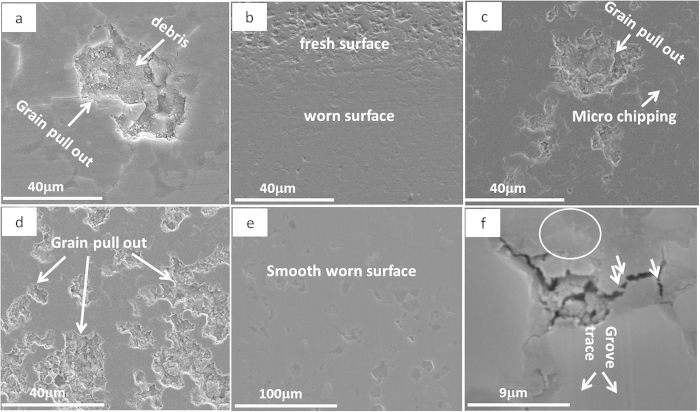
SEM images of (**a**) pure Al_2_O_3_, (**b**) S_0.5-0_, (**c**) S_2-0_, (d) S_5-0_, (**e**) S_0.3-1_, and (**f**) higher resolution image (e).

To understand the role of GNTs in the wear mechanism, 5 different samples with various GNT contents were chosen and their wear tracks were further investigated by using SEM, compared with wear tracks of the pure Al_2_O_3_, and the results are shown in Figs 9 and 10. All the selected wear tracks were subject to the same sliding loads, for comparison purpose. As described earlier, the surface of pure Al_2_O_3_ is unsmooth, with large islands of pull-out grains (Fig. 9a). Within the grains, a large amount of debris was visible at higher resolution, Fig. 10a shows the coarse wear behaviour discussed above. Adding 0.5 wt% of GNP alone into the Al_2_O_3_ made the wear track narrower (Fig. 9b) than that of the pure Al_2_O_3_, and a smooth tribofilm appeared on the worn surface, as shown in Fig. 10b. This smooth tribofilm decreased the wear friction between the sample and the counterpart ball, thus improved the wear resistance, as shown in Fig. 6. Further GNP addition up to 2 wt% led to a decreased area of the smooth tribofilm (Fig. 9c), and micro chipping with grain pull-outs also became visible on the worn surface (Fig. 10c), compared with S_0.5-0_. The deteroiated wear resistance for S_5-0_ (Fig. 9d) corresponded to the large areas of intergranular grain pull-out, as shown in Figs 9d and 10d, due to its poor mechaninal properties as listed in Table 1. The wear track became narrowest, even narrower in S_0.3-1_ than S_0.5-0_ (Fig. 9e), corresponding to the highest amount of coherent tribofilm (Fig. 10e). These study confirmed the key relationship between the GNT content and the formation of a tribofilm during wear, and helped to explain why the S_0.3-1_ exhibited even better wear resistant behavior than the S_0.5-0_ as presented in Fig. 8.

Further, the fracture toughness of S_0.3-1_ is slightly higher than that of S_0.5-0_, due to different roles of GNPs and CNTs in toughening the composites, as discussed previously^11^. Easier CNT de-bundling in the presence of GNPs is another advantage of the hybrid reinforcement agent in S_0.3-1_, which helped the well-dispersed CNTs to better exhibit their merit in the composite, than them working alone.

**Figure 11:**
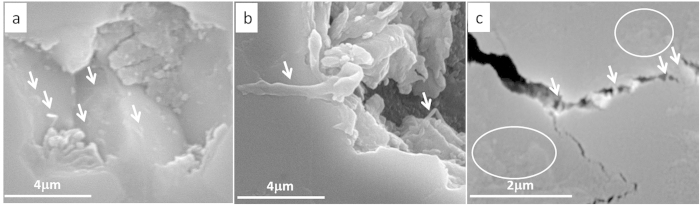
High resolution SEM images from S_0.3-1_ showing (**a**) embedded CNTs, (**b**) embedded GNPs on the top of a worn surface, (**c**) CNT bridging cracked grains (arrowed) and GNP lying on the worn surface (circled).

The reduced grain pull-outs could be a result of less tangential frictional forces between the ball and the composite surface, due to formation of a protective tribofilm by the GNT exfoliation on the wear surface. Fig. 11a–c provides evidence for the direct role of GNTs in the formation of the protective tribofilm during the wear test. The embedded GNTs from unpolished (ground only) surface will be exposed and spread on the wear track during the reciprocating movements, to form the tribofilm (Fig. 11a,b). The flattened GNTs on the worn surface are clearly visible in Figs 10f and 11c (circled), which could be the feeding stock for the tribofilm. GNPs are likely to contribute more effectively to the tribofilm than that of CNTs, due to their layered and their easy to be exfoliated structures; whilst CNTs’ rolling effect, along such tribofilm, cannot be ignored in the reduction of COF and wear rates^24^.

**Figure 12:**
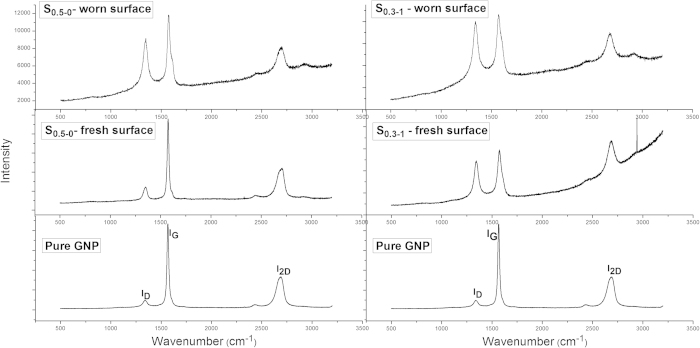
The comparison of Raman scans of pure GNP and Al_2_O_3_-GNT composites obtained from surfaces with and without wear.

Further, the existence of CNTs indirectly contributed more in bridging the grains against crack propagations (due their higher aspect ratio) in case of micro-chipping and grain pull-outs, by improving the mechanical properties of the composites (Figs 10f and 11c). In fact, samples without CNTs but with higher GNP contents (S_2-0_ and S_5-0_) drastically degraded the wear resistant property (Fig. 6), because of the poor mechanical properties (Table1) which ended up with severe grain pull-outs (Fig. 9c,d). Therefore, both the lubricating film and the improved mechanical properties together improved the wear resistant properties of the composites. Furthermore, although sufficient amounts of flattened CNTs on the wear track could indeed lubricate the surface, as reported in our previous work on Al_2_O_3_-5 wt%CNT composites^24^, the fact that very low GNP contents (S_0.5-0_ and S_0.3-1_) in the composites could lead to the formation of tribofilms suggests the dominant role of GNPs in the improved tribological performance in this context. The existence of the fragmented GNPs during tribology testing is confirmed by our Raman studies, as shown in Fig. 12.

Two types of areas on S_0.5-0_ and S_0.3-1_ were chosen during our Raman scanning: 1) surfaces away from wear damaged areas (*i.e* no influence of the wear); and 2) surfaces inside the wear tracks which were subject to the entire wear process. Compared with the pure GNP Raman spectra, three typical peaks were observed at 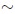
1350 cm^−1^ (D band), 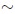
1585 cm^−1^ (G band) and 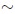
2700 cm^−1^ (2D band) in the fresh surfaces for S_0.5-0_ and S_0.3-1_^25^, confirming that there is no damage to GNPs during the sintering process. Inside the wear track of S_0.5-0_, the scans revealed an increase in the D peak intensity. The increased D peak intensity is directly related to the number of edges, corresponding to more GNP flakes in this context, given the uniform GNP dispersion in the as-synthesised composites. Thus, the increased I_D_/I_G_ ratios inside the wear track of S_0.5-0_ indeed confirmed the formation of extra fragmented GNP flakes during the tribology testing. However, the exfoliation and fragmented flakes were believed to be the source for the tribofilm formation. Similar behaviour has also been observed by other authors for silicon nitride-GNP composites^14^,^17^. The increases in the I_D_/I_G_ ratios in worn surfaces of S_0.3-1_ are also obvious, indicating the exfoliation and tribofilm formation, even at very low GNP content. Another interesting point about the Al_2_O_3_-GNT composites is that such tribofilms could be maintained during the entire wear process, as eventually damaged tribofilms on the surface can continuously be replaced or regenerated by the embedded GNTs inside the Al_2_O_3_ matrix.

## Conclusion

The tribological properties of the hybrid GNT reinforced-Al_2_O_3_ composites were investigated using a ball-on-disc technique. Samples designated as S_0.5-0_ and S_0.3-1_ showed a remarkable 70% and 86% reduction in the wear rates and 23% and 20% reduction in COF values, respectively, against the pure Al_2_O_3_, under 15 N sliding load. It was identified that the superior mechanical traits of the S_0.5-0_ and S_0.3-1_ samples, in term of fracture toughness, against pure Al_2_O_3_ and the formation of a protective tribofilm on the wear track are the effective wear mechanisms for converting Al_2_O_3_-GNT composites into wear resistant materials. The CNTs played a vital indirect role in the former, whilst GNPs contributed directly to the latter tribofilm formation which is more dominant for the reduced COF. These newly developed novel hybrid composites, possessing promising toughness, machinability and tribological performance, could extend their application to many new fields as advanced structural materials, protective coatings for micro-mechanical systems and contact-damage-resistant components.

## Additional Information

**How to cite this article**: Yazdani, B. *et al* Tribological performance of Graphene/Carbon nanotube hybrid reinforced Al_2_O_3_ composites. *Sci. Rep* **5**, 11579; doi: 10.1038/srep11579 (2015).

## Figures and Tables

**Table 1:** A summary of the relative densities, mechanical properties and brittleness index of the hot-pressed pure Al_2_O_3_ and nanocomposite samples.

**Material**	**Sample ID (S**_**X-Y**_)	**Relative density (%)**	**Hardness Vickers (GPa)**	**Flexural strength (MPa)**	**SENB Fracture toughness (MPa.m**^**1/2**^)	**Brittleness Index (BI)**
Pure Al_2_O_3_	S_0-0_	98	15.9	369	3.5	4.5
Al_2_O_3_-0.5 wt% GNP	S_0.5-0_	99.2	15.6	390	5.5	2.83
Al_2_O_3_-2 wt% GNP	S_2-0_	98	7.5	296	3.9	1.92
Al_2_O_3_-5 wt% GNP	S_5-0_	97	4.2	120	2.7	1.5
Al_2_O_3_-1 wt% GNP+1 wt% CNT	S_1-1_	99	11.2	270	3.5	3.14
Al_2_O_3_-0.3 wt% GNP+1 wt% CNT	S_0.3-1_	99	16	430	5.8	2.7
